# Image synthesis of monoenergetic CT image in dual‐energy CT using kilovoltage CT with deep convolutional generative adversarial networks

**DOI:** 10.1002/acm2.13190

**Published:** 2021-02-18

**Authors:** Daisuke Kawahara, Shuichi Ozawa, Tomoki Kimura, Yasushi Nagata

**Affiliations:** ^1^ Department of Radiation Oncology Institute of Biomedical & Health Sciences Hiroshima University Hiroshima Japan; ^2^ Hiroshima High‐Precision Radiotherapy Cancer Center Hiroshima Japan

**Keywords:** deep learning, artificial Intelligence, dual‐energy CT, image synthesis

## Abstract

**Purpose:**

To synthesize a dual‐energy computed tomography (DECT) image from an equivalent kilovoltage computed tomography (kV‐CT) image using a deep convolutional adversarial network.

**Methods:**

A total of 18,084 images of 28 patients are categorized into training and test datasets. Monoenergetic CT images at 40, 70, and 140 keV and equivalent kV‐CT images at 120 kVp are reconstructed via DECT and are defined as the reference images. An image prediction framework is created to generate monoenergetic computed tomography (CT) images from kV‐CT images. The accuracy of the images generated by the CNN model is determined by evaluating the mean absolute error (MAE), mean square error (MSE), relative root mean square error (RMSE), peak signal‐to‐noise ratio (PSNR), structural similarity index (SSIM), and mutual information between the synthesized and reference monochromatic CT images. Moreover, the pixel values between the synthetic and reference images are measured and compared using a manually drawn region of interest (ROI).

**Results:**

The difference in the monoenergetic CT numbers of the ROIs between the synthetic and reference monoenergetic CT images is within the standard deviation values. The MAE, MSE, RMSE, and SSIM are the smallest for the image conversion of 120 kVp to 140 keV. The PSNR is the smallest and the MI is the largest for the synthetic 70 keV image.

**Conclusions:**

The proposed model can act as a suitable alternative to the existing methods for the reconstruction of monoenergetic CT images in DECT from single‐energy CT images.

## 1 INTRODUCTION

In a conventional kilovoltage computed tomography (kV‐CT) image, the value of a pixel represents the photon attenuation of the tissue in that pixel. However, materials with similar absorbances have the same CT numbers. Therefore, distinguishing between these materials becomes challenging; for example, it is difficult to distinguish between iodinated contrast medium and hemorrhage.^1^, ^2^

Dual‐energy computed tomography (DECT) is based on the fact that x‐ray attenuation depends primarily on the photoelectric effect and Compton scattering in the diagnostic energy range, and that these attenuation phenomena are energy dependent.^3^ DECT scans are acquired using two different tube potentials, which can be used to estimate the Compton scattering and the photoelectric effect components of the attenuation. Subsequently, this information is used to distinguish between tissues and characterize materials. Using this technique, the monoenergetic CT number, effective atomic number, urinary stone characterization, and virtual noncontrast‐enhanced images may be reconstructed.^4^ The monoenergetic CT image can be reconstructed at an energy level ranging from 40 to 140 keV.^5^ Furthermore, it can achieve better soft‐tissue contrasts for radiotherapy treatment planning and radiation diagnosis by reducing the effect of image artifacts resulting from the presence of metal.^6^ The GE Revolution CT scanner with Gemstone Spectral Imaging (GSI) allows for dual energy kV‐CT acquisitions that can be used to generate monoenergetic, iodine contrast‐enhanced, calcium‐enhanced, and effective atomic number images.^7^ The disadvantages of DECT are that it requires a higher radiation dose and more expensive than conventional multidetector CT.

Image synthesis with deep learning is used for image‐to‐image translation from magnetic resonance (MR) images to CT images and for multicontrast MR images with convolutional neural networks (CNNs).^8^ CNNs can capture and represent high‐dimensional input–output relationships. CNNs have been applied to medical image segmentation and computer‐aided detection.^9^ Florkow et al. designed a two‐dimensional (2D) CNN model that generates a synthetic CT images from a T1‐weighted MR images. In the kV‐CT images synthesized from MR images, a large difference with variations of up to 17% was observed in terms of the mean absolute error and variations of up to 28% specifically in bone images. These differences are attributable to the spatial resolution of MR images being poorer than that of CT images.^10^ The current study proposes the image synthesis of monoenergetic CT images from kV‐CT images, both of which have the same resolution.

Recently, an image synthesis method based on a generative neural network (GAN) has been used. The GAN based on the CNN model operates by training two different networks: a generator network to synthesize an image and a discriminator network to distinguish between synthesized and reference images.^11^ Herein, the synthesis of monoenergetic CT images at 40, 70, and 140 keV from equivalent kV‐CT images using the GAN model is proposed.

## 2 MATERIALS AND METHODS

### 2.1 Data acquisition

A total of 18,084 images from 28 patients were analyzed as part of an institutional review board‐approved study. DECT images for each patient were acquired using the Revolution DECT scanner (GE Healthcare, Princeton, NJ, USA). DECT acquisitions at 80 and 140 kV tube voltages and an exposure of 560 mA were performed. The other scanning parameters were a field‐of‐view of 360 mm, slice thickness of 0.5 mm, and rotation time of 1.0 s. The monoenergetic CT images at 40, 70, and 140 keV and the equivalent kV‐CT images were reconstructed using GSI and defined as the reference images.

### 2.2 Deep learning model

**Fig. 1:**
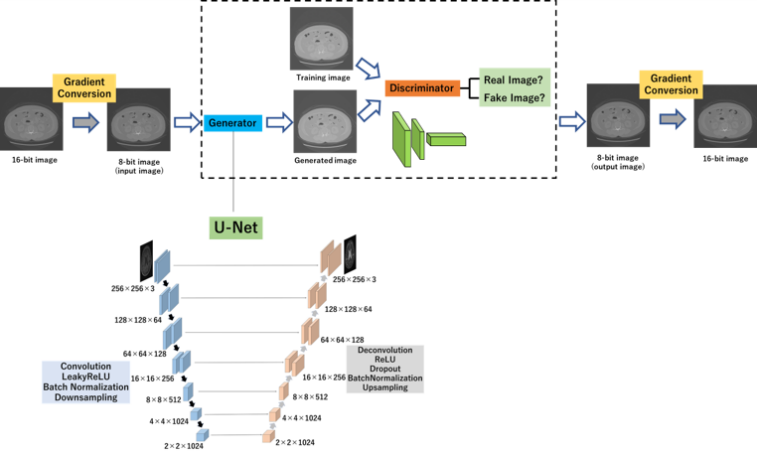
GAN framework. Generator learns to generate monoenergetic CT images of an anatomy similar to the kV‐CT images. Meanwhile, discriminator learns to discriminate between the synthetic and real monoenergetic CT images.

In the current study, a 2D CNN model comprising a GAN was designed. An overview of the GAN network model is depicted in Fig. 1. It includes a generator to estimate the monoenergetic CT image and a discriminator to distinguish the real monoenergetic CT image from the generated one. The generator attempts to produce realistic images that confuses the discriminator. Notably, these two networks are trained simultaneously. Hyperparameter optimization was performed in the training dataset, and the test set settings were adjusted only once for each algorithm. Image red–green–blue (RGB) channels are typically used as inputs to the neural network.^12^ A 16‐bit DICOM image was converted to an 8‐bit RGB portable network graphics (PNG) image, and the output 8‐bit RGB PNG image from the 2D CNN model was converted to 16‐bit DICOM images. The pixel number in the CT image ranged from −1000 to 3079 Hounsfield units (HU). Subsequently, the values of the pixel in CT images were converted to 8‐bit (0–255) images by dividing with 16, which is the value obtained by dividing the maximum pixel value, that is, 3079 HU, by 256. In this study, monoenergetic CT images were generated at 40, 70, and 140 keV from an equivalent kV‐CT image at 120 kVp.

**Fig. 2:**
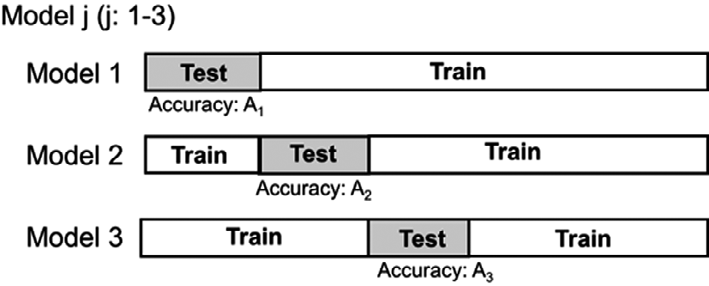
Generation and testing of the prediction model. Model performance was evaluated via cross‐validation.

**Fig. 3:**
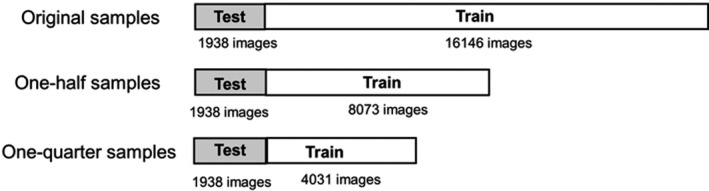
Evaluation of the predictive model based on the number of samples. Training dataset was reduced to one‐half and one‐quarter.

The dataset of 18,084 images comprised DECT images scanned from the feet to the chest of 28 patients. The data were categorized into two sets: 16146 images (20 patients) for model training and 1938 images (8 patients) for model testing. The training–testing processes were repeated three times for cross‐validation, as depicted in Fig. 2. Furthermore, the performance of the predictive model was evaluated with the training data reduced to one‐half and one‐quarter; this was done to reduce the instabilities with respect to changes in the number of samples, as depicted in Fig. 3.

The proposed models were implemented using TensorFlow packages (V1.7.0, Python 2.7, CUDA 9.0) on a Ubuntu 16.04 LTS system. All the three models were trained using instance normalization and identical hyperparameters except for the batch size. In the instance normalization, the mean and standard deviation were calculated and normalized across each channel in each training example. At each iteration, a minibatch of 2D images was randomly selected from the training set. The batch size was limited by the graphics processing unit (GPU) memory. Three hundred epochs were used to operate the 2D model on an 11‐GB NVIDIA GeForce GTX 1080 GPU.

### 2.3 Evaluation

The prediction accuracy of the model for synthetic and real monoenergetic CT images were evaluated using the following five metrics: relative mean absolute error (MAE), relative root mean square (RMSE), structural similarity index (SSIM), peak signal‐to‐noise ratio (PSNR), and mutual information (MI). These metrics are defined as follows:

(1) $$MAE=\frac{1}{n_{x}n_{y}}\sum_{i,j}^{n_{x}n_{y}}\frac{\left|r\left(i,j\right)‐t\left(i,j\right)\right|}{r\left(i,j\right)}$$

Here, $r\left(i,j\right)$ is the value of pixel $\left(i,j\right)$ in the synthetic CT image, $t\left(i,j\right)$ is the value of pixel $\left(i,j\right)$ in the reference image, and $n_{x}n_{y}$ is the total number of pixels. RMSE is defined as

(2) $$RMSE=\sqrt{\frac{1}{n_{x}n_{y}}\sum_{i,j}^{n_{x}n_{y}}{\left(\frac{r\left(i,j\right)‐t\left(i,j\right)}{r\left(i,j\right)}\right)}^{2}}$$

The SSIM is discrete form, as follows, and luminance to compute a similarity score between two images.

The SSIM between two images $\vec{x}$ and $\vec{y}$ can be computed as Ref. [13].

(3) $$SSIM\left(\vec{x},\vec{y}\right)=\frac{\left(2\mu_{x}\mu_{y}+C_{1}\right)\left(2\sigma_{\mathrm{xy}}+C_{2}\right)}{\left(\mu_{x}^{2}+\mu_{x}^{2}+C_{1}\right)\left(\sigma_{x}^{2}+\sigma_{y}^{2}+C_{2}\right)}$$

(4) $$C_{1}={\left(k_{1}Q\right)}^{2},\;k_{1}=0.01$$

(5) $$C_{2}={\left(k_{2}Q\right)}^{2},\;k_{2}=0.03$$

$C_{1}$ and $C_{2}$ are constants that are used to prevent a zero denominator and to maintain the stability of the formula. Q is the maximum CT value for the synthetic and reference images. The values of $k_{1}$ and $k_{2}$ are generally obtained from Ref. [14]. $\sigma_{x}$ is an estimate in the discrete form, as follows.

(6) $$\sigma_{x}={\left(\frac{1}{N‐1}\sum_{i=1}^{N}{\left(x_{i}‐\mu_{x}\right)}^{2}\right)}^{1/2}$$

The correlation coefficient between $\vec{x}$ and $\vec{y}$ is denoted as $\sigma_{\mathrm{xy}}$, which is expressed as follows.

(7) $$\sigma_{\mathrm{xy}}=\frac{1}{N‐1}\sum_{i=1}^{N}\left(x_{i}‐\mu_{x}\right)\left(y_{i}‐\mu_{y}\right),$$

and $\mu_{x}$ is the mean intensity and can be expressed as

(8) $$\mu_{x}=\frac{1}{N}\sum_{i=1}^{N}x_{i}.$$

The PSNR is calculated as follows:

(9) $$PSNR_{\mathrm{GL}}=10\times log_{10}\left(\frac{{\left(\mathrm{MAX}\right)}^{2}}{\mathrm{MSE}}\right).$$

Here, MAX and MSE are the possible maximum signal intensity and the mean square error (or difference) of the image, respectively. The MI is used as a cross‐modality similarity measure^15^ and is calculated as follows:

(10) $$I\left(r:t\right)=\sum_{m\in I_{r}}\sum_{n\in I_{t}}p\left(m,n\right)log\left(\frac{p\left(m,n\right)}{p\left(m\right)p\left(n\right)}\right).$$

**Fig. 4:**
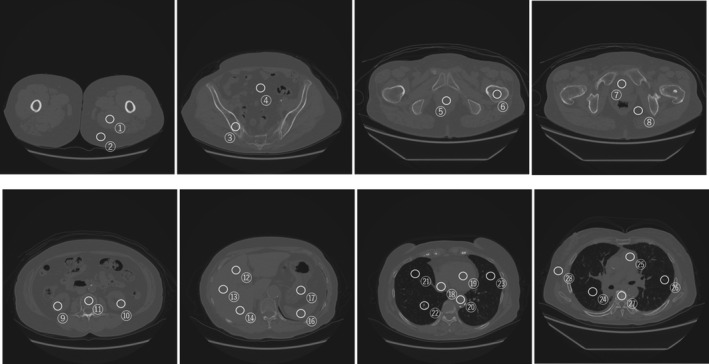
Measurement of the monoenergetic CT number from the feet‐to‐chest slice. Average and SD values of the monoenergetic CT number were measured by creating a circular ROI of 2 cm in diameter.

Here, m and n are the intensities in the reference monoenergetic CT image *I_r_* and synthesized monoenergetic CT image *I_t_*, respectively. *p(m, n)* is the joint probability density of *I_r_* and *I_t_*, whereas *p(m)* and *p(n)* are marginal densities. Furthermore, *p(m, n)* can be calculated as follows:

(11) $$p\left(m,n\right)=\frac{h\left(m,n\right)}{\sum_{m\in I_{t}}\sum_{n\in I_{t}}h\left(m,n\right)},$$

where $h\left(m,n\right)$ is the histogram of the pixel values in the reference monoenergetic CT image *I_r_* and synthesized monoenergetic CT image *I_t_*. Furthermore, the difference in the synthesized and reference monoenergetic CT numbers in the region of interest (ROI) was evaluated for several slices, starting from the feet to the chest in a manually drawn ROI, as depicted in Fig. 4

## 3 RESULTS

**Fig. 5:**
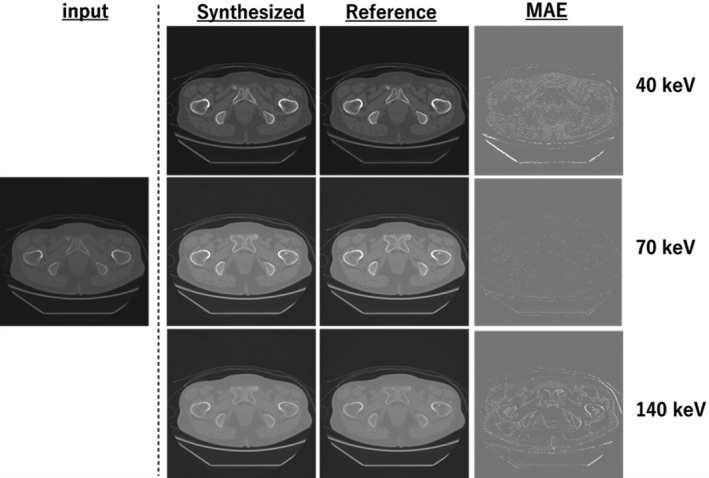
Samples obtained from monoenergetic CT image generation at the pelvic level: input image is the equivalent kV‐CT image, synthetic and reference images are the monoenergetic CT images at 40, 70, and 140 keV, and MAE is the difference between the synthesized and reference monoenergetic CT images.

**Fig. 6:**
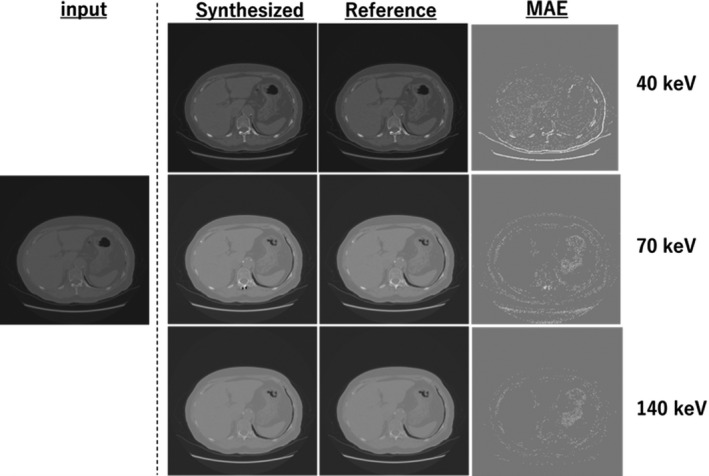
Samples obtained from monoenergetic CT image generation at the abdominal level: input image is the equivalent kV‐CT image, synthetic and reference images are the monoenergetic CT images at 40, 70, and 140 keV, and MAE is the difference between the synthesized and reference monoenergetic CT images.

**Fig. 7:**
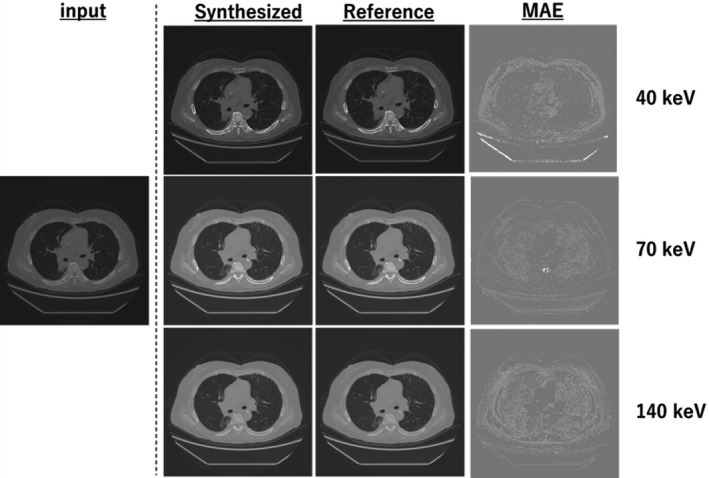
Samples obtained from monoenergetic CT image generation at the chest level: input image is the equivalent kV‐CT image, synthetic and reference images are the monoenergetic CT images at 40, 70, and 140 keV, and MAE is the difference between the synthesized and reference monoenergetic CT images.

Figures 5, 6, 7 show the samples obtained by cross‐modality generation for the synthetic monoenergetic CT images at 40, 70, and 140 keV. Table 1 presents the difference in the monoenergetic CT numbers between the synthetic and reference images. As can be seen, the difference is within 9.8 HU at 40 keV, −16.4 HU at 70 keV, and −7.6 HU at 140 keV. Furthermore, the difference in the monoenergetic CT numbers in the ROIs between the synthetic and reference images was within the appropriate range of the SD values.

**Table 1:** Difference (Δ) and SD values of the monoenergetic CT number between the synthetic and reference monoenergetic CT images at 40, 70, and 140 keV for Model 1.

40 keV			70 keV			140 keV		
	Δ(HU)	SD (HU)		Δ(HU)	SD (HU)		Δ(HU)	SD (HU)
①	9.82	19.15	①	‐5.26	9.32	①	‐1.90	7.38
②	6.17	20.09	②	‐5.03	8.15	②	‐1.15	5.10
③	3.06	15.26	③	‐7.40	7.78	③	‐2.09	8.92
④	3.61	70.49	④	‐3.61	31.51	④	‐1.19	16.31
⑤	‐3.17	21.42	⑤	‐6.74	12.94	⑤	4.76	11.01
⑥	0.39	23.84	⑥	‐8.95	14.75	⑥	2.05	7.85
⑦	‐7.61	24.12	⑦	‐2.89	9.82	⑦	‐1.52	6.89
⑧	‐7.70	23.18	⑧	‐5.95	9.71	⑧	0.81	6.88
⑨	4.00	17.62	⑨	4.00	17.62	⑨	‐7.01	7.02
⑩	‐0.65	18.07	⑩	‐0.65	18.07	⑩	1.97	6.81
⑪	0.02	58.49	⑪	0.02	58.49	⑪	4.00	11.10
⑫	3.38	15.22	⑫	‐3.58	9.12	⑫	‐2.34	7.39
⑬	4.79	16.10	⑬	‐6.90	8.87	⑬	‐0.18	6.02
⑭	‐4.59	17.44	⑭	‐8.12	9.18	⑭	1.05	5.15
⑮	‐3.46	43.44	⑮	‐5.93	20.75	⑮	‐1.06	12.82
⑯	0.30	15.68	⑯	‐3.12	8.63	⑯	‐1.73	5.34
⑰	‐6.44	10.55	⑰	‐6.44	10.55	⑰	‐7.38	6.91
⑱	‐1.72	12.34	⑱	‐1.72	12.34	⑱	‐7.62	7.91
⑲	5.30	12.54	⑲	5.30	12.54	⑲	‐7.02	8.81
⑳	‐3.03	42.98	⑳	‐3.03	42.98	⑳	‐6.11	19.27
㉑	7.54	182.01	㉑	7.54	182.01	㉑	‐6.92	197.10
㉒	‐7.00	51.98	㉒	‐7.00	51.98	㉒	‐5.42	80.94
㉓	0.26	37.22	㉓	‐8.84	93.06	㉓	0.19	96.82
㉔	‐6.58	12.61	㉔	‐4.26	8.70	㉔	2.04	5.38
㉕	‐3.96	12.61	㉕	‐6.33	18.09	㉕	‐2.35	14.11
㉖	‐4.45	72.94	㉖	‐16.71	33.37	㉖	5.34	19.97
㉗	0.23	13.89	㉗	‐8.48	8.72	㉗	‐1.60	5.34

Tables 2, 3, 4 lists the average MAE, MSE, RMSE, PSNR, and MI from the feet‐to‐chest slice for synthetic monoenergetic CT images at 40, 70, and 140 keV. There were no significant differences between the image synthesis performance in cross‐validation from the results of the MAE, MSE, RMSE, PSNR, SSIM, and MI. Moreover, there were no significant differences between image synthesis performance due to the number of samples from the result of the MAE, MSE, RMSE, PSNR, SSIM, and MI. The MAE, MSE, and RMSE were the smallest for the synthetic monoenergetic CT image at 140 keV and the largest for the synthetic monoenergetic CT image at 70 keV. The PSNR was the smallest for the synthetic monoenergetic CT image at 70 keV and largest for the synthetic monoenergetic CT image at 140 keV. The MI was the largest for the synthetic monoenergetic CT image at 70 keV and smallest for the synthetic monoenergetic CT image at 40 keV.

**Table 2:** Average MAE, MSE, RMSE, PSNR, and SSIM values computed from the feet‐to‐chest slice for synthetic monoenergetic CT images at 40 keV using cross‐validation, and validation by changing number of samples.

	MAE		MSE		RMSE		PSNR		SSIM		MI	
	Average	SD	Average	SD	Average	SD	Average	SD	Average	SD	Average	SD
Model 1	0.023	0.002	0.004	0.002	0.058	0.018	39.992	2.81	0.988	0.001	2.299	0.151
Model 2	0.025	0.003	0.004	0.000	0.062	0.009	39.281	1.232	0.987	0.002	2.263	0.183
Model 3	0.022	0.002	0.004	0.002	0.057	0.011	39.729	2.625	0.989	0.001	2.293	0.142
One‐half data	0.022	0.001	0.004	0.000	0.059	0.011	39.652	1.344	0.988	0.001	2.255	0.188
One‐quarter data	0.023	0.002	0.004	0.002	0.060	0.012	39.832	1.352	0.988	0.001	2.229	0.174

**Table 3:** Average MAE, MSE, RMSE, PSNR, and SSIM computed from feet‐to‐chest slice for synthetic monoenergetic CT images at 70 keV with cross‐validation, and validation by changing number of samples.

	MAE		MSE		RMSE		PSNR		SSIM		MI	
	Average	SD	Average	SD	Average	SD	Average	SD	Average	SD	Average	SD
Model 1	0.015	0.003	0.001	0.001	0.026	0.005	46.753	1.605	0.994	1	2.185	0.112
Model 2	0.016	0.004	0.001	0.000	0.028	0.003	45.921	0.912	0.993	1.920	2.151	0.136
Model 3	0.014	0.003	0.001	0.001	0.025	0.003	46.445	1.499	0.995	1.000	2.179	0.105
One‐half data	0.014	0.002	0.001	0.000	0.027	0.003	46.355	1.344	0.994	0.670	2.144	0.139
One‐quarter data	0.015	0.003	0.001	0.002	0.028	0.004	46.411	1.352	0.988	0.892	2.142	0.132

**Table 4:** Average MAE, MSE, RMSE, PSNR, and SSIM computed from feet‐to‐chest slice for synthetic monoenergetic CT images at 140 keV with cross‐validation, and validation by changing number of samples.

	MAE		MSE		RMSE		PSNR		SSIM		MI	
	Average	SD	Average	SD	Average	SD	Average	SD	Average	SD	Average	SD
Model 1	0.022	0.003	0.002	0.001	0.041	0.010	42.842	2.098	0.988	0.001	1.936	0.099
Model 2	0.024	0.004	0.002	0.000	0.044	0.005	42.080	0.920	0.987	0.002	1.906	0.120
Model 3	0.021	0.003	0.002	0.001	0.040	0.006	42.560	1.960	0.989	0.001	1.931	0.093
One‐half data	0.021	0.002	0.002	0.000	0.042	0.006	42.478	1.198	0.988	0.001	1.899	0.123
One‐quarter data	0.022	0.003	0.002	0.001	0.042	0.005	42.472	1.232	0.988	0.001	1.901	0.112

The time required to create the image synthetic model was approximately 154.8 ± 3.2 h for image synthesis. The times to create the synthetic monoenergetic CT images using all the trained models were 7.8–8.2 images/s.

## 4 DISCUSSION

Zhao et al. developed a deep learning model to map low‐ to high‐energy images using a two‐stage CNN. They evaluated the virtual noncontrast (VNC) imaging reconstructed by DECT from the kV‐CT image scanned using single‐energy CT (SECT).^15^ The difference in the monoenergetic CT numbers between the predicted and original high‐energy CT images was below 4.0 HU in the abdominal region. In this regard, the current study proposes a prediction model for the generation of monoenergetic CT images from kV‐CT images using GAN in the feet‐to‐chest region. The difference in the monoenergetic CT numbers in the ROIs between the synthetic and reference images ranged from −16.4 to 9.82 HU, which was within the SD. Furthermore, in the conversion of a gray level image from 16‐bit to 8‐bit color, the SD value used was 16 HU; this value was also within the SD range in a phantom with 120‐kV CT images. Our proposed model successfully yielded highly accurate DECT images in the presence of noise from the kV‐CT images. The standard deviation values vary with the scan mAs used, but were not considered in the current study. To highlight the prediction performance, the relationship of the mAs, the SD, and the accuracy of the image synthesis is needed to investigate.

The current study used 8‐bit PNG image converted from 16‐bit DICOM image for the deep learning model. The images in PNG format can be utilized by any deep learning framework.^16^ However, the image conversion of the 16‐bit to 8‐bit has a potentially merit and demerit. A decrease scale resolution from 2‐byte images such as 16‐bit per pixel to one‐byte such as 8‐bit per pixel usually implies a halving of the digital storage space needed for the image. On the other hand, a decrease scale resolution causes that important diagnostic information may be lost in the process. Smith et al reported that a 2‐byte to 1‐byte reduction in gray scale resolution may be done without significant loss of diagnostic information for MR images.^17^ The future work will be performed to evaluate of the image quality and lesion detectability due to the difference of the scale resolution in the SECT and DECT images.

The monoenergetic CT image at 140 keV indicated the smallest values for MAE, MSE, and RMSE but the largest for PSNR and SSIM. In the monoenergetic CT image at a high energy, the contrast scale in the monoenergetic CT number from low to high density was smaller than that at a lower energy. It is therefore easier to predict the pixel values for small contrast scales at high energies. Notably, the MAE, MSE, RMSE, PSNR, and SSIM values were found to be dependent on the contrast scale with monoenergetic CT images.

The MI with the monoenergetic CT image at 70 keV was the largest. Yu et al. reported that the effective energies of 80 and 140 kV were in the range 59.8–81.5 keV.^18^ The monoenergetic CT image at 70 keV, which is close to the effective x‐ray energy at 120 kV, contained pixel information similar to that with a continuous energy of 120 kV. Meanwhile, the monoenergetic CT image at 70 keV indicated the smallest MAE, MSE, RMSE, PSNR, and SSIM. As presented in Table 2, the average difference in the monoenergetic CT numbers between the synthesized and reference monoenergetic CT images was −0.42 HU at 40 keV, −4.45 at 70 keV, and −1.57 HU at 140 keV. This suggests that the image prediction model with the monoenergetic CT image at 70 keV generated a systematic error. Further studies involving an increase in the number of epochs or modification of the model architecture are required.

The current study demonstrates that highly accurate monoenergetic CT images can be generated from kV‐CT images using a GAN model employing a deep learning approach. Our proposed model is therefore capable of generating monoenergetic CT images at energies ranging from low to high, that is, from 78 to 82 s for 640 kV‐CT images in a single sequence. The lower energies offer better contrasts for soft tissues, whereas higher energies reduce the beam hardening artifacts, thereby further enhancing the material decomposition accuracy.

In the current study, 120 kV‐CT images reconstructed via DECT was used. Tawfil et al. investigated the difference in the image quality of 120 kV‐CT images scanned via SECT and the equivalent 120 kV‐CT images reconstructed via DECT based on the data of clinical patients.^19^ Notably, the subjective image quality scores between the DECT and SECT groups did not indicate a significant difference. Hence, DECT images can be obtained from SECT images using the proposed model. As such, the method can significantly improve the scan time, thereby reducing the scanning dose and imaging cost as well as improving the efficiency of analysis, such as segmentation and image registration. For radiation diagnosis, monoenergetic CT images should assist in lesion detectability.

Recently, concerns regarding the application of deep learning in medical images have been reported. More samples used may yield poorer performances. In the current study, cross‐validation was performed, and the effect of using different number of samples on image synthesis was evaluated. It was discovered that the number of samples did not significantly affect the image quality of the synthesized images. However, small perturbations may result in severe artifacts in the reconstruction. Furthermore, an equivalent kV‐CT image at 120 kVp reconstructed from DECT images via GSI was used in the current study. It was discovered that no perturbation occurred by the body movement and respiratory motion. However, perturbation in the sampling domain by the inevitable noise dominated or equipment malfunction may occur. This can generate a myriad of different artifacts. Furthermore, failure to reconstruct a small structural change may occur. Hence, the image quality should be evaluated in the lesion or in each organ. This is a limitation of the current study; therefore, it will be performed in a future study.

## 5 CONCLUSION

Synthetic medical image generation can be a cost‐saving approach for developing automated diagnostic technology. The image prediction framework of a kV‐CT image equivalent to a monoenergetic CT image was proposed. It is expected that the proposed model can serve a suitable alternative to the existing methods for the reconstruction of monoenergetic CT images in DECT from SECT images.

## Informed consent

Informed consent was obtained from all individual participants included in the study.

## Ethnical approval

All procedures performed in studies involving human participants were in accordance with the ethical standards of the institutional and/or national research committee and with the 1964 Helsinki declaration and its later amendments or comparable ethical standards.

## Conflict of Interest

The author have no other relevant of conflict of interest to disclose.

## Advances in knowledge

We created a new image prediction model for monoenergetic CT images scanned from kV‐CT images.
